# Pretreatment neutrophil-to-lymphocyte ratio predicts clinical relapse of ulcerative colitis after tacrolimus induction

**DOI:** 10.1371/journal.pone.0213505

**Published:** 2019-03-07

**Authors:** Yu Nishida, Shuhei Hosomi, Hirokazu Yamagami, Naoko Sugita, Shigehiro Itani, Tomomi Yukawa, Koji Otani, Yasuaki Nagami, Fumio Tanaka, Koichi Taira, Noriko Kamata, Tetsuya Tanigawa, Toshio Watanabe, Yasuhiro Fujiwara

**Affiliations:** Department of Gastroenterology, Osaka City University Graduate School of Medicine, Osaka, Japan; Kurume University School of Medicine, JAPAN

## Abstract

**Objectives:**

Although tacrolimus is useful as an induction therapy in patients with ulcerative colitis (UC), information regarding the long-term outcome after tacrolimus therapy is insufficient. The aim of this study was to evaluate the clinical significance of the pretreatment neutrophil-to-lymphocyte ratio (NLR) as a prognostic factor in patients with UC receiving tacrolimus, to aid treatment selection.

**Materials and methods:**

Patients with moderate-to-severe active UC who received oral tacrolimus induction therapy and subsequent immunomodulatory maintenance therapy at our hospital between 2009 and 2017 and who showed clinical response at week 12, were retrospectively enrolled. Cox regression analysis was conducted to study the prognostic role of the pretreatment NLR. The combined impact of the NLR and other known prognostic factors was investigated with multivariate regression.

**Results:**

Among 45 patients included in this study, 21 patients experienced relapse during a median follow-up period of 16.6 months. Multivariate Cox regression analysis identified the pretreatment NLR (hazard ratio [HR]: 0.82, 95% confidence interval [CI]: 0.72–0.94, *P* < 0.01) and the use of immunomodulators at the start of tacrolimus treatment (HR: 0.18, 95% CI: 0.05–0.66, *P* = 0.01) as independent predictors of clinical relapse.

**Conclusions:**

The pretreatment NLR is an independent prognostic factor in patients with UC treated with tacrolimus.

## Introduction

Ulcerative colitis (UC) is a type of inflammatory bowel disease affecting the colorectum, with an unknown etiology. Corticosteroids are effective in inducing remission of UC; however, approximately 20–30% of cases are refractory to or dependent on corticosteroid therapy.[1, 2] In such cases, treatment with anti-tumor necrosis factor (TNF) agents (i.e., infliximab, adalimumab, or golimumab) or calcineurin inhibitors (i.e., cyclosporine or tacrolimus) has been recommended.[3–5] Infliximab administration is well established as both an induction therapy and a maintenance therapy in patients with UC. Nevertheless, although randomized controlled trials have demonstrated the efficacy of oral tacrolimus for remission induction therapy for patients with UC, [3, 6] immunomodulators (IMs) are recommended for maintaining remission after the induction therapy.[5] In fact, the long-term outcome after tacrolimus treatment has been disappointing, [7] and poor relapse-free survival and long-term colectomy-free rates have been reported.[8, 9] However, the risk factors for clinical relapse after tacrolimus therapy have not been sufficiently analyzed.

The neutrophil-to-lymphocyte ratio (NLR) is an easily accessible laboratory test and has been reported as a useful predicting factor for various types of cancer, [10–13] rheumatoid arthritis[14] and coronary heart disease.[15, 16] Several studies have reported the existence of a relationship between the NLR and the disease activity of UC.[17, 18] There has been a previous report on the utility of the pretreatment NLR as a useful marker for predicting the loss of response to infliximab in patients with UC.[19] Namely, a high NLR is strongly and independently associated with an increased risk of loss of response to infliximab in patients with UC.[19] However, the clinical utility of the pretreatment NLR for patients with UC receiving tacrolimus has not been analyzed. Therefore, the objective of this study was to evaluate the clinical significance of the pretreatment NLR as a prognostic factor in patients with UC receiving tacrolimus, which would aid in treatment selection.

## Materials and methods

### Patients

All patients with moderate-to-severe active UC, who started oral tacrolimus treatment at our hospital between January 2009 and December 2017, were retrospectively enrolled. As the aim of this study was to evaluate the prognostic value of the NLR for long-term outcome, patients who were nonresponsive to tacrolimus by week 12 were excluded. Patients who were not receiving IMs for maintaining remission at the time of withdrawal of tacrolimus or those receiving anti-TNF agents were also excluded.

The diagnosis of UC was based on clinical, endoscopic, and histopathologic findings. Demographic, clinical, and laboratory data were obtained from the medical records. The differential white blood cell count was analyzed using an XE-5000 hematology analyzer (Sysmex, Kobe, Japan), according to the manufacturer’s protocol. In each case, the NLR was calculated from a blood sample by dividing the absolute neutrophil count with the absolute lymphocyte count.

Patients were followed up from the time of tacrolimus administration to clinical relapse, cessation of IM maintenance therapy, loss to follow-up, or until the end of October 2018.

### Tacrolimus therapy

The initial dosage of oral tacrolimus administration was 0.05 mg/kg twice per day. Blood tacrolimus levels were measured 2 or 3 times per week for the first 2 weeks of tacrolimus initiation. Doses were adjusted to achieve a high target trough level of 10–15 ng/mL. After maintaining high trough levels for 2 weeks, the dose was decreased to achieve a low trough level target of 5–10 ng/mL. The duration of tacrolimus administration is officially limited up to 12 weeks because of the absence of long-term data about the efficacy and safety of this regimen. Tacrolimus is withdrawn or continued according to clinical requirements under the discretion of the physician, and IMs are usually started as a maintenance therapy before the withdrawal of tacrolimus.

### Study endpoints

The primary outcome measure of this study was the clinical relapse of UC.

### Definitions

The partial Mayo (p-Mayo) score[20] was used to assess clinical disease activity. Moderate-to-severe active disease was defined as a p-Mayo score of ≥4. Severe UC was defined as a p-Mayo score of ≥7. Clinical relapse was defined as an exacerbation of gastrointestinal symptoms that required secondary alternative therapies such as surgery, corticosteroids, or anti-TNFα drugs. Clinical response was defined as a p-Mayo score reduction of ≥3 points, accompanied by a decrease of at least 30% from the baseline and a decrease in the rectal bleeding subscore of ≥1 or an absolute rectal bleeding subscore of 0 or 1.[21] The pretreatment NLR was calculated from a blood sample obtained within 1 week before the start of tacrolimus. Post-treatment NLR was defined at approximately 12 week after the start of tacrolimus.

The steroid-refractory state was defined as the presence of active disease despite either intravenous prednisolone at >1 mg·kg^-1^·day^-1^ for at least 1 week or oral prednisolone at >30 mg/day for at least 2 weeks. The steroid-dependent state was defined either the inability to taper prednisolone to <10 mg/day without disease recurrence or relapse within 3 months of stopping prednisolone.

### Statistical analysis

Continuous variables were summarized as medians and interquartile ranges. The differences in clinical characteristics were compared using either the chi-square test or Fisher’s exact test for categorical variables and the Mann-Whitney U-test for continuous variables. Correlations were calculated using Spearman’s rank correlation. Differences in the pretreatment and post-treatment NLR were compared using the Wilcoxon rank sum test. Receiver operating characteristic (ROC) curves were plotted to calculate the area under the ROC curve. The cumulative relapse-free rate was illustrated using a Kaplan-Meier plot. Differences in the survival curves were assessed with the log-rank test. Furthermore, continuous values of laboratory data were evaluated with the Cox proportional hazard model. Data were presented as hazard ratios (HRs) with 95% confidence intervals (CIs). Multivariate Cox regression analyses were performed to identify factors associated with clinical relapse; those factors speculated to be risk factors of clinical relapse were then entered into a multivariate analysis.

A *P*-value of <0.05 was considered statistically significant. All statistical analyses were performed with EZR (Saitama Medical Center, Jichi Medical University), a graphical user interface for R (The R Foundation for Statistical Computing, version 2.13.0). More precisely, it is a modified version of R commander (version 1.6–3) that includes statistical functions frequently used in biostatistics.

### Ethical considerations

This study was approved by Osaka City University Hospital Certified Review Board; (no. 3569), which waived the requirement for written informed consent because the analysis used anonymized clinical data that were retrospectively obtained after each patient agreed to receive the treatment. Nevertheless, all patients were notified of the content and information of this study and given the opportunity to refuse participation. None of the patients refused participation. This study followed the Ethical Guidelines for Medical and Health Research Involving Human Subjects established by the Ministry of Education, Culture, Sports, Science and Technology and the Ministry of Health, Labor and Welfare in Japan.

## Results

### Study subjects

**Fig 1:**
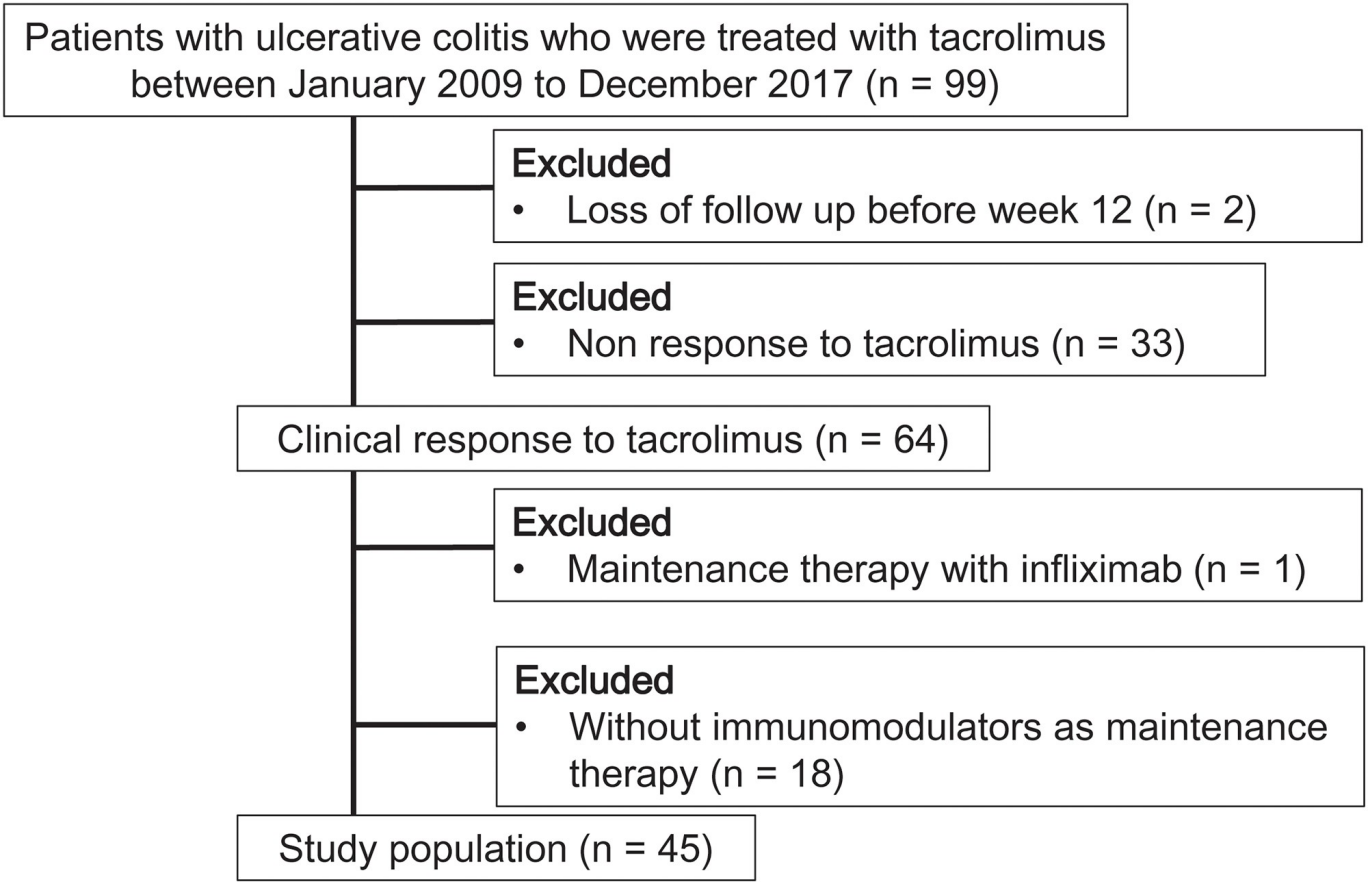
Details of study selection.

In the present study, a cohort of 99 patients with UC treated with oral tacrolimus was screened. Among them, 2 patients were excluded because of loss to follow-up before week 12 and 33 patients were excluded for being nonresponsive to tacrolimus. Sixty-four patients (66.0%) were considered responders. Of those, 1 responder was excluded for receiving infliximab as a maintenance therapy and 18 patients were excluded for not receiving IMs as a maintenance therapy at the time of tacrolimus withdrawal. Finally, 45 patients were enrolled in this study (Fig 1). During a median follow-up period of 16.6 months (interquartile range: 6.9–54.6 months), 21 of 45 patients experienced relapsed. The demographic characteristics of the patients are summarized in Table 1. One patient was excluded from post-treatment NLR analysis because he did not present a differential white blood cell count at around 12 weeks. The NLR decreased after tacrolimus induction therapy in both patients with sustained response (*P* < 0.01) and patients with clinical relapse (*P* < 0.05), using the Wilcoxon rank sum test (S1 Fig).

**Table 1:** Baseline characteristics of the study population and comparison between the low NLR group and high NLR group.

	all patients	low NLR group	high NLR group	*P*-value
Number of patients	45	23	22	
Gender: male/female	26 / 19	11/12	15/7	0.231
Age at diagnosis (years), median (interquartile range)	32.2 (23.1–39.8)	33.2 (24.9–38.2)	31.1 (23.1–45.0)	0.785
Age at start of tacrolimus (years), median (interquartile range)	36.7 (28.8–49.3)	36.7 (29.2–46.6)	40.6 (28.6–53.0)	0.454
Disease duration (years), median (interquartile range)	2.7 (1.2–8.6)	3.2 (2.1–6.0)	1.8 (0.7–10.1)	0.454
UC location: Left-sided colitis/Pancolitis	11 / 34	5/18	6/16	0.738
Response to corticosteroids				
Dependent, n (%)	19 (42.2%)	10 (43.5%)	9(40.9%)	1
Resistant, n (%)	22 (48.9%)	9 (39.1%)	13 (59.1%)	0.238
Concomitant therapies at start of tacrolimus, n (%)				
Mesalamine	41 (91.1%)	21 (91.3%)	20 (90.9%)	1
Corticosteroids	35 (77.8%)	14 (60.9%)	21 (95.5%)	< 0.01
Dosage of corticosteroids (mg/day), median (interquartile range)	30.0 (5.0–40.0)	10.0 (0.0–40.0)	30.0 (16.3–57.5)	0.051
partial Mayo score, median (interquartile range)	7 (6–7)	7 (6–7.5)	7 (6–7)	0.595
Presence of CMV antigenemia, n (%)	4 (8.9)	1 (4.3)	3 (13.6)	0.346
Hemoglobin (g/dl), median (interquartile range)	12.0 (10.8–13.1)	11.8 (10.1–12.8)	12.4 (11.1–13.3)	0.323
Albumin (g/dl), median (interquartile range)	3.20 (2.70–3.60)	3.30 (2.75–3.55)	3.20 (2.78–3.72)	0.955
CRP (mg/dl), median (interquartile range)	2.25 (0.41–6.11)	3.30 (0.34–6.76)	2.21 (0.80–5.63)	0.937
WBC (/μL), median (interquartile range)	9400 (6500–11400)	7300 (5700–10400)	10500 (8600–11800)	0.01
Neutrophil (/μL), median (interquartile range)	7300 (6900–8400)	5700 (3700–7600)	8900 (7100–10000)	< 0.01
Lymphocyte (/μL), median (interquartile range)	1200 (800–1500)	1400 (1200–2000)	900 (600–1100)	< 0.01
pretreatment NLR, median (interquartile range)	5.84 (3.25–9.45)	3.25 (2.70–4.44)	9.63 (7.64–13.20)	< 0.01
post-treatment NLR, median (interquartile range)	2.50 (1.57–4.52)	2.30 (1.36–3.70)	3.05 (1.83–4.90)	0.222
Clinical relapse, n (%)	21 (46.7%)	14 (60.9%)	7 (31.8%)	0.075

CMV: cytomegalovirus; WBC: white blood cell; CRP: C-reactive protein; NLR: neutrophil to lymphocyte ratio.

### Risk factors for relapse

The patients’ background, concomitant therapy, and laboratory data before tacrolimus therapy were analyzed to identify risk factors for relapse after tacrolimus therapy. According to univariate Cox regression analysis, relapse-free survival exhibited a significant relationship with the pretreatment NLR (HR: 0.89, 95% CI: 0.80–1.00, *P* < 0.05), which was mainly because of lymphocytes rather than neutrophils (Table 2). In contrast, it did not exhibit a significant relationship with the post-treatment NLR (HR: 0.98, 95% CI: 0.89–1.06, *P* = 0.75) (Table 2). Multivariate Cox regression analyses were performed to identify factors associated with clinical relapse; those factors speculated to be risk factors of clinical relapse were then entered into a multivariate analysis. The multivariate analysis identified the NLR (HR: 0.82, 95% CI: 0.72–0.94, *P* < 0.01) and the use of IMs at the start of tacrolimus (HR: 0.18, 95% CI: 0.05–0.66, *P* = 0.01) as independent prognostic factors for clinical relapse (Table 2).

**Table 2:** Cox regression analysis of the risk for relapse during follow-up after induction of tacrolimus.

	Unadjusted HR (95% CI)	*P*-value	Adjusted HR (95% CI)	*P*-value
Gender				
Male	1			
Female	0.81 (0.33–1.97)	0.63		
Age at diagnosis (continuous, per 10 years old)	1.04 (0.75–1.43)	0.81		
Age at start of tacrolimus (continuous, per 10 years old)	0.95 (0.69–1.30)	0.76		
Disease duration (continuous)	0.95 (0.88–1.03)	0.27		
Disease location				
left-sided colitis	1			
pan-colitis	1.67 (0.49–5.71)	0.41		
Prednisolone dependent				
No	1			
Yes	0.83 (0.34–2.01)	0.68		
Prednisolone resistant				
No	1			
Yes	1.61 (0.68–3.84)	0.28		
Mesalamine treatment at start of tacrolimus				
No	1			
Yes	0.66 (0.15–2.84)	0.57		
Steroid treatment at start of tacrolimus				
No	1		1	
Yes	1.03 (0.37–2.81)	0.96	1.40 (0.46–4.29)	0.55
Immunmodulators (azathioprine or 6-mercaptopurine) at start of tacrolimus				
No	1		1	
Yes	0.33 (0.10–1.14)	0.08	0.18 (0.05–0.66)	0.01
Severe ulcerative colitis (partial Mayo score ≥ 7) at start of tacrolimus				
No	1			
Yes	0.85 (0.36–2.02)	0.71		
albumin (continuous)	1.06 (0.44–2.58)	0.89	1.59 (0.64–3.98)	0.32
CRP (continuous)	0.99 (0.90–1.08)	0.75		
Pretreatment NLR (continuous)	0.89 (0.80–1.00)	0.045	0.82 (0.72–0.94)	< 0.01
Neutrophil (continuous, per 1000 /μL)	0.97 (0.86–1.10)	0.64		
Lymphocyte (continuous, per 1000 /μL)	2.17 (1.33–3.56)	< 0.01		
Post-treatment NLR (continuous)	0.98 (0.89–1.09)	0.75		

CRP: C-reactive protein; CI: confidence interval; HR: hazard ratio; NLR: neutrophil to lymphocyte ratio.

**Fig 2:**
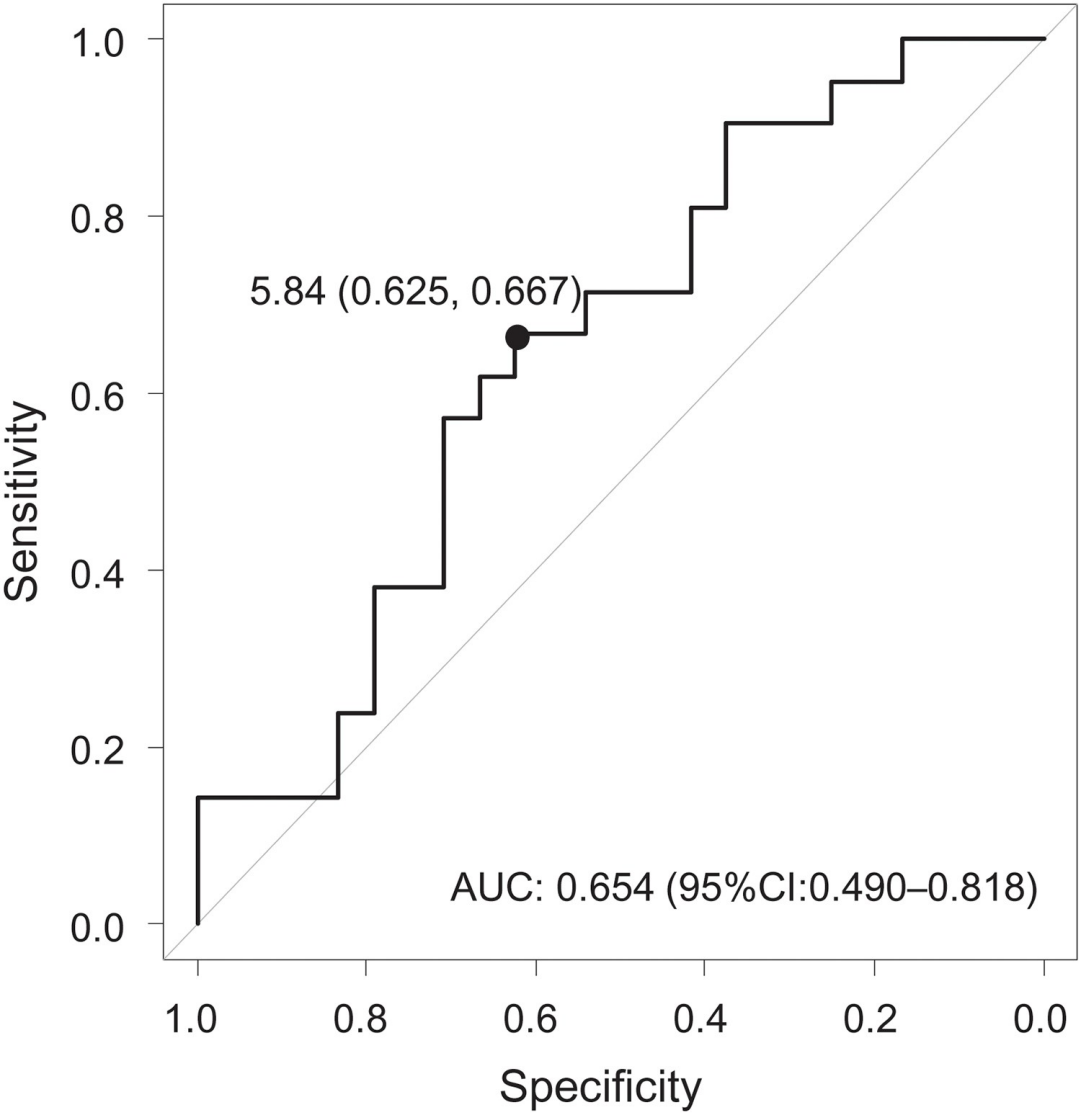
Receiver operating characteristic curve for determining the cutoff value of the pretreatment neutrophil-to-lymphocyte ratio (NLR) for predicting clinical relapse in patients with ulcerative colitis (UC). Area under curve (AUC): 0.65 (95% confidence interval [CI]: 0.49–0.82).

When the NLR was examined as a dichotomous variable, a cutoff value of the NLR for the risk of relapse was determined using ROC analysis. The ROC analysis showed that the best cutoff value for the NLR was > 5.84 (sensitivity: 62.5%, specificity: 66.7%) (Fig 2). Therefore, the cutoff value of 5.84 was chosen for further study. Twenty-three (51.1%) patients had an NLR of < 5.84 at pretreatment (low NLR group), whereas 22 (48.9%) patients had an NLR of ≥ 5.84 (high NLR group). Table 1 shows a comparison of the baseline characteristics between the low NLR group and the high NLR group. The percentage of patients with concomitant corticosteroid use was higher in the high NLR group. No significant differences were detected between the high NLR group and the low NLR group in other clinical variables.

**Fig 3:**
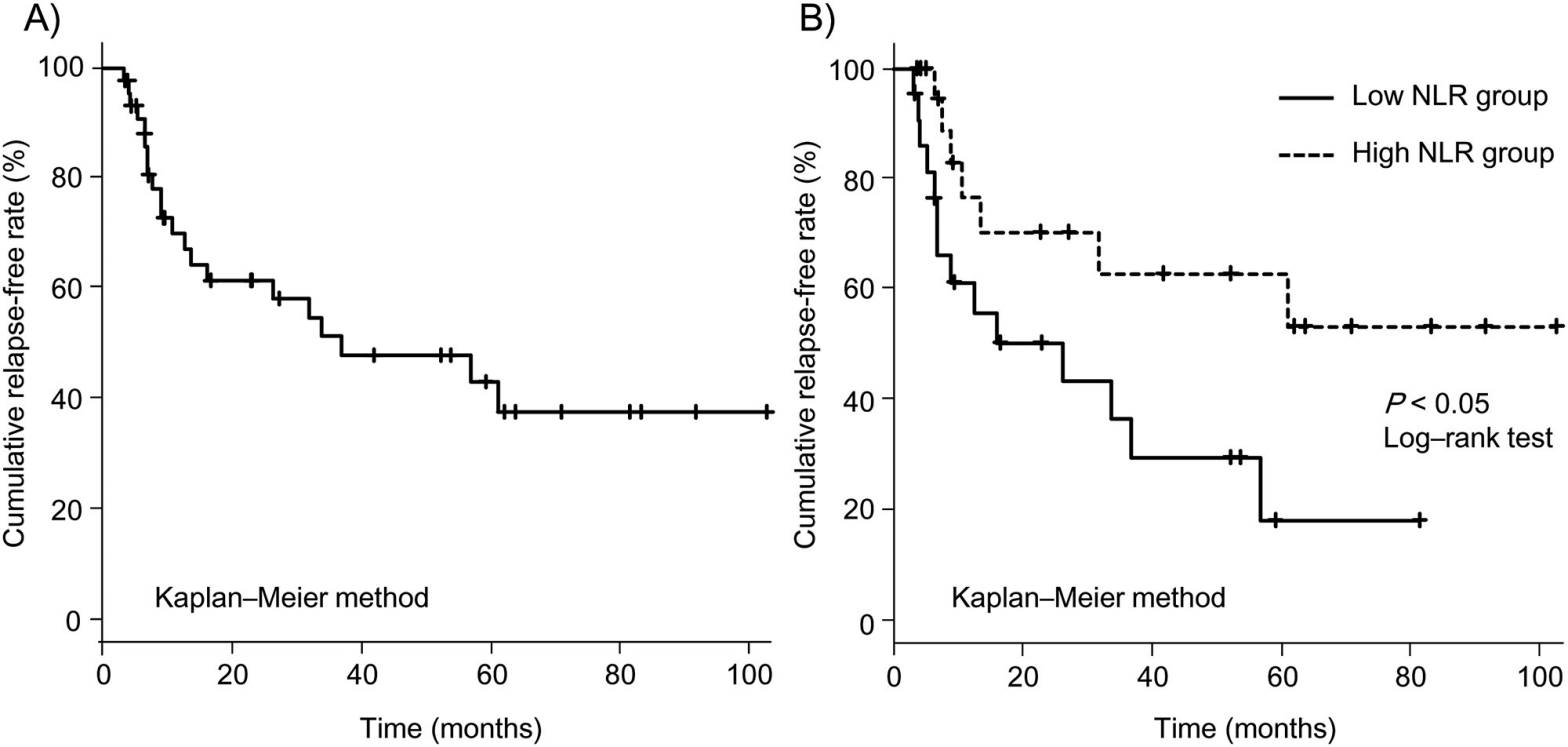
Relapse-free survival after tacrolimus therapy. Overall relapse-free survival in 45 responders to tacrolimus (A) and relapse-free survival based on the neutrophil-to- lymphocyte ratio (NLR). The overall survival rate was significantly better in the high NLR group (*P* < 0.05, log-rank test) (B).

In the low NLR group, 60.9% (14 of 23) of the patients experienced relapse during the follow-up period. In the high NLR group, 31.8% (7 of 22) of the patients experienced relapsed. Fig 3 shows the overall relapse-free survival of all responders and the relapse-free survival based on the NLR. The overall survival rate was significantly better in the high NLR group (*P* < 0.05, log-rank test; Fig 3B).

### Influence of concomitant therapy on the NLR

**Fig 4:**
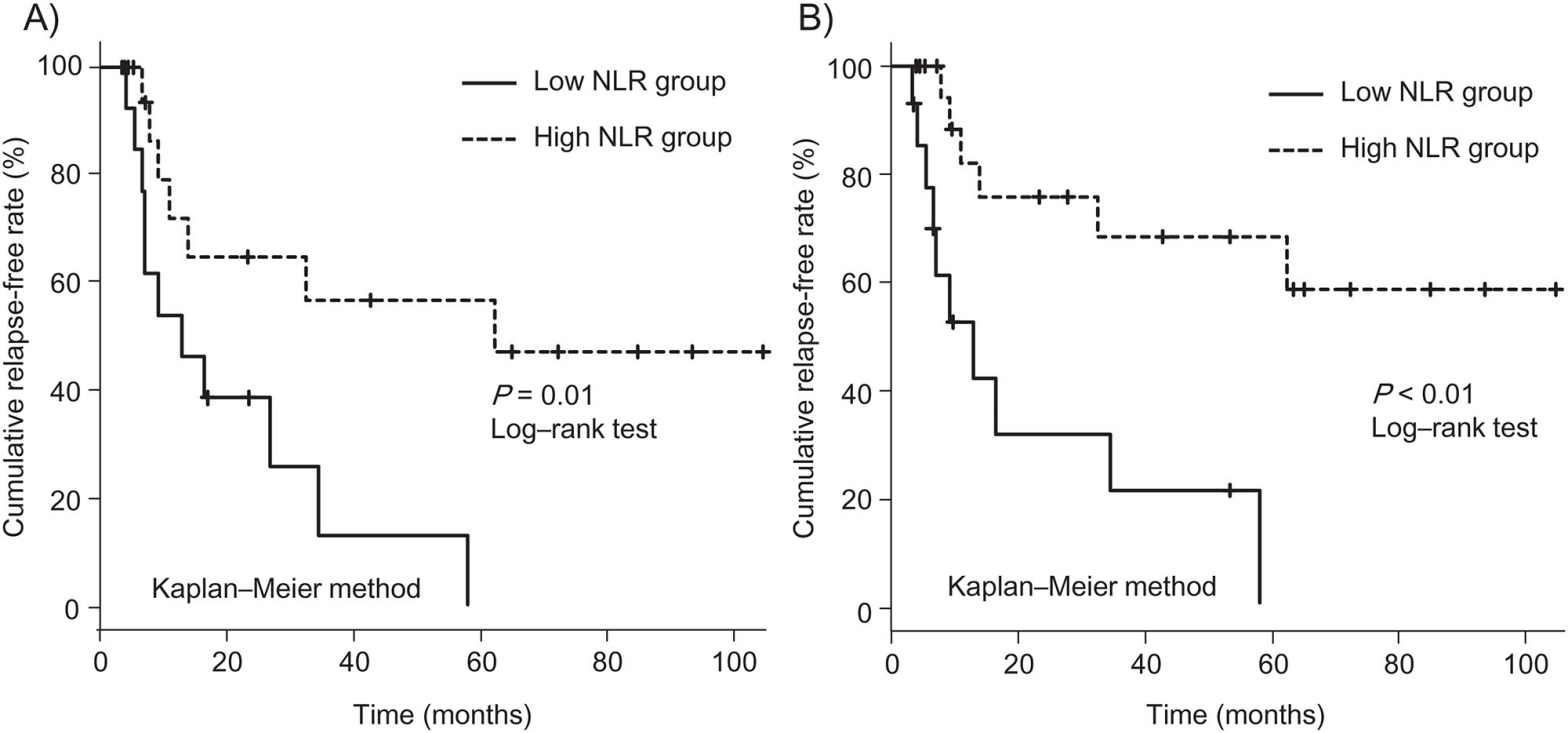
Relapse-free survival after tacrolimus therapy based on concomitant therapy. Relapse-free survival in patients without immunomodulator use at the start of tacrolimus (A) and in patients with corticosteroid use at the start of tacrolimus (B). The overall survival rate was significantly better in those with a high neutrophil-to-lymphocyte ratio (NLR) in each group.

Concomitant therapies and corticosteroid response state may affect the NLR level as a prognostic factor after tacrolimus therapy. However, the median pretreatment NLR was 5.26 (interquartile range: 3.31–7.84) in corticosteroid dependent patients and 7.52 (interquartile range: 3.86–11.9) in corticosteroid resistant patients. No significant difference was noted between these two groups (*P* = 0.429). Patients using corticosteroid had higher NLR values mainly because of higher neutrophil counts, and patients using IM had lower NLR values mainly because of lower neutrophil counts (Table 3), whereas no correlation was noted between corticosteroid dosage and the pretreatment NLR (*r* = 0.0199, *P* = 0.91) (S2 Fig). Furthermore, the use of IMs before the start of tacrolimus was an independent prognostic factor for clinical relapse (Table 2). The influence of concomitant therapies before starting tacrolimus should be considered. Therefore, we next conducted a subgroup analysis based on the use of IMs or corticosteroids. As tacrolimus is usually administered for steroid-refractory patients, most patients in this study did not receive IMs but received corticosteroids. We performed a subgroup analysis for patients who did not receive IMs at the start of tacrolimus (n = 32, who had a tendency to have higher NLRs) and patients with corticosteroid use at the start of tacrolimus (n = 35, whose NLRs were significantly higher than those of patients without corticosteroid use) (Table 3). Fig 4 shows the Kaplan-Meir plot for clinical relapse in each group, and the relapse-free rate was significantly better in patients with a high NLR in each group. In multivariate Cox regression analysis, the NLR was also identified as an independent prognostic factor for clinical relapse in each group (patients without IM use, HR: 0.83, 95% CI: 0.72–0.95, *P* < 0.01; patients with corticosteroid use, HR: 0.82, 95% CI: 0.71–0.94, *P* < 0.01). With respect to patients with IM use or patients without corticosteroid use, we could not identify the NLR as a statistically significant prognostic factor in each group, possibly because of the small number of the patients.

**Table 3:** Comparison of baseline characteristics based on concomitant immunomodulators or corticosteroids.

	Without IM	With IM	*P*-value
WBC (/μL), median (interquartile range)	10400 (8600–11800)	6500 (4700–7500)	0.001
Neutrophil (/μL), median (interquartile range)	8200 (6400–9500)	4800 (3000–6200)	0.001
Lymphocyte (/μL), median (interquartile range)	1200 (900–1600)	1000 (800–1300)	0.409
NLR, median (interquartile range)	7.00 (4.17–10.97)	3.64 (2.98–5.93)	0.064
Low NLR group / High NLR group	14/18	9/4	0.189
Clinical relapse, n (%)	18 (56.2)	3 (23.1)	0.055
	Without PSL	With PSL	*P*-value
WBC (/μL), median (interquartile range)	6000 (4400–8000)	9800 (7900–11800)	0.004
Neutrophil (/μL), median (interquartile range)	4000 (3000–5500)	7800 (6200–9500)	0.001
Lymphocyte (/μL), median (interquartile range)	1200 (800–1500)	1100 (800–1500)	0.512
NLR, median (interquartile range)	2.93 (2.53–3.58)	7.12 (4.62–10.44)	0.001
Low NLR group / High NLR group	9/1	14/21	0.010
Clinical relapse, n (%)	5 (50.0)	16 (45.7)	1

IM: immunomodulator; PSL: corticosteroid; WBC: white blood cell; NLR: neutrophil to lymphocyte ratio.

### Discussion

This is the first study to describe the utility of the pretreatment NLR in patients with UC receiving tacrolimus therapy. The principal finding of this study was that a low pretreatment NLR is strongly and independently associated with an increased risk of clinical relapse after tacrolimus induction therapy for UC. As the NLR cutoff value of 5.84 was a predictor of relapse, patients with low NLR should be followed up with caution and given optimal maintenance therapy.

To date, the underlying mechanism of the association of the NLR with the clinical course of UC remains poorly understood. The NLR represents the balance between systematic inflammatory response and immune response. This study showed that clinical relapse after tacrolimus therapy was associated with the NLR in patients with UC who received IMs as a maintenance therapy. That is, 13 patients received IMs at the start of tacrolimus and the remaining 32 patients received IMs as additional maintenance treatment before the withdrawal of tacrolimus. Therefore, the NLR may predict the efficacy of IMs as a maintenance therapy as UC relapses during IM maintenance therapy after the withdrawal of tacrolimus. In this study, the prognostic value of the NLR could be largely due to lymphocytes (Table 2). Azathioprine is a prodrug that enters the endogenous purine salvage pathway to be converted to its active end-product, thioguanine nucleotides (TGNs), which are incorporated into DNA and inhibit T-cell activation signals.[22–25] Several studies have reported the association between leukocyte counts or leukocyte subsets and IM efficacy, [26–29] and the mechanism of immune suppression by IMs is relevant to lymphocytes.[25] Smith et al. reported that lymphocyte counts were significantly different when patients with TGNs exceeding the target range were compared with those with undetectable TGN levels, and TGN levels do correlate with response to thiopurine treatment.[29, 30] Conversely, Fraser et al. reported that lymphocyte count had no value for predicting the induction of remission.[28] However, to our knowledge, no study has evaluated the value of lymphocytes as predictor of maintenance therapy. Our study may suggest that IMs are more effective for patients with low baseline lymphocyte counts. From this view, the NLR may also be useful for selecting the maintenance therapy after not only tacrolimus but also other induction therapies such as corticosteroids or cytapheresis. Further studies would be needed to assess the utility of the NLR for other therapies.

Concomitant therapies may affect the pretreatment NLR and clinical relapse after tacrolimus therapy. Although no correlation was noted between corticosteroid dosage and the pretreatment NLR (S2 Fig), patients receiving corticosteroids at the start of tacrolimus had a significantly higher pretreatment NLR, and those receiving IMs at the start of tacrolimus had a significantly lower NLR; however, the association between the NLR and the relapse-free survival rate was independent of concomitant corticosteroid use or concomitant IM use. Furthermore, we performed a subgroup analysis to elucidate the influence of concomitant therapies based on the use of concomitant corticosteroids or concomitant IMs. In both the corticosteroid group and the non-IM group, the NLR was identified as an independent prognostic factor for clinical relapse even in this subgroup analysis.

The appropriate therapies for steroid-refractory UC remain controversial. The European Crohn’s and Colitis Organization (ECCO) guidelines equally recommend the use of tacrolimus and infliximab for patients with intravenous steroid-refractory UC, [5] and many studies have compared the efficacy of tacrolimus and infliximab in terms of short-term and long-term outcomes[31–34]; however, their results were not consistent and the choice of treatment remains controversial. The choice of treatment for steroid-refractory UC depends on the physician’s decision. It has been previously reported that a high pretreatment NLR was risk factor of loss of response to infliximab, [19] whereas the current study showed that a high pretreatment NLR is a protective factor against clinical relapse after tacrolimus therapy. These studies suggest that patients with a high NLR should be preferably treated with tacrolimus and patients with a low NLR should be preferably treated with infliximab. Further studies comparing treatments with tacrolimus and infliximab would be needed to determine the utility of NLR for treatment selection.

In this study, we also identified the use of IMs at the start of tacrolimus as an independent prognostic factor for clinical relapse (Table 3). Thiopurines are slow-acting drugs, [35] taking up to 6 months to reach a therapeutic effect. Patients who started IMs just before tacrolimus withdrawal would experience relapse before the treatment takes effect. Although the ECCO guidelines recommend IM maintenance therapy after tacrolimus induction therapy, [5] the timing of the introduction of IM is not defined. Our findings suggest that IMs should be started as soon as possible after deciding to start tacrolimus therapy.

This study has some limitations. First, this is a retrospective study with relatively small cohort, which is susceptible to bias in data selection and analysis. Second, the NLR differs among patients and can be influenced by concurrent infection and concomitant drugs. Although we usually perform fecal culture to rule out infectious colitis or cytomegalovirus (CMV) antigenemia before starting tacrolimus, infectious colitis or bacterial translocation or viral infection may be involved in the disease state. We could not estimate the association between other viruses and NLR or the disease state and could not eliminate the possibility that such infections may have influenced the results. With respect to concomitant drugs, we considered the influence of concomitant corticosteroids or IMs. It would be preferable to analyze the predictive value of NLR by dividing the patients into 4 groups, as of presence / absence of corticosteroid and presence / absence of corticosteroid. However, in this study, the sample size was rather small, it was difficult to analyze the predictive value of NLR by dividing into these 4 groups. Furthermore, other drugs such as mesalamine may also affect the NLR or disease course. Further, as we do not routinely measure fecal calprotectin, which may be a prognostic factor of UC, or procalcitonin levels, we could not take into account other biomarkers. Therefore, further large prospective studies will help confirm the NLR as a key predictor in tacrolimus treatment for UC.

Despite these limitations, our study suggests that the pretreatment NLR could be associated with clinical relapse after tacrolimus therapy in patients with UC and should be introduced in clinical practice. Taking NLR into account may aid the treatment selection for patients with UC.

## Supporting information

S1 Fig Time course of the neutrophil-to-lymphocyte ratio (NLR). The NLR decreased after tacrolimus induction therapy in both patients with sustained response (*P* < 0.01) (A) and patients with clinical relapse (*P* < 0.05) (B) (the Wilcoxon rank sum test).
(TIF)

S2 Fig Scatter plot of the pretreatment neutrophil-to-lymphocyte ratios (NLR) and corticosteroid dosage among patients with corticosteroid. There was no correlation between corticosteroid dosage and the pretreatment NLR (r = 0.0199, *P* = 0.91) (the Spearman’s rank correlation).
(TIF)
