# Supplementary figures and images for: Pretreatment neutrophil-to-lymphocyte ratio predicts clinical relapse of ulcerative colitis after tacrolimus induction
Source: PLoS One. 2019 Mar 7;14(3):e0213505. doi: 10.1371/journal.pone.0213505 (PMC6405082; doi:10.1371/journal.pone.0213505)

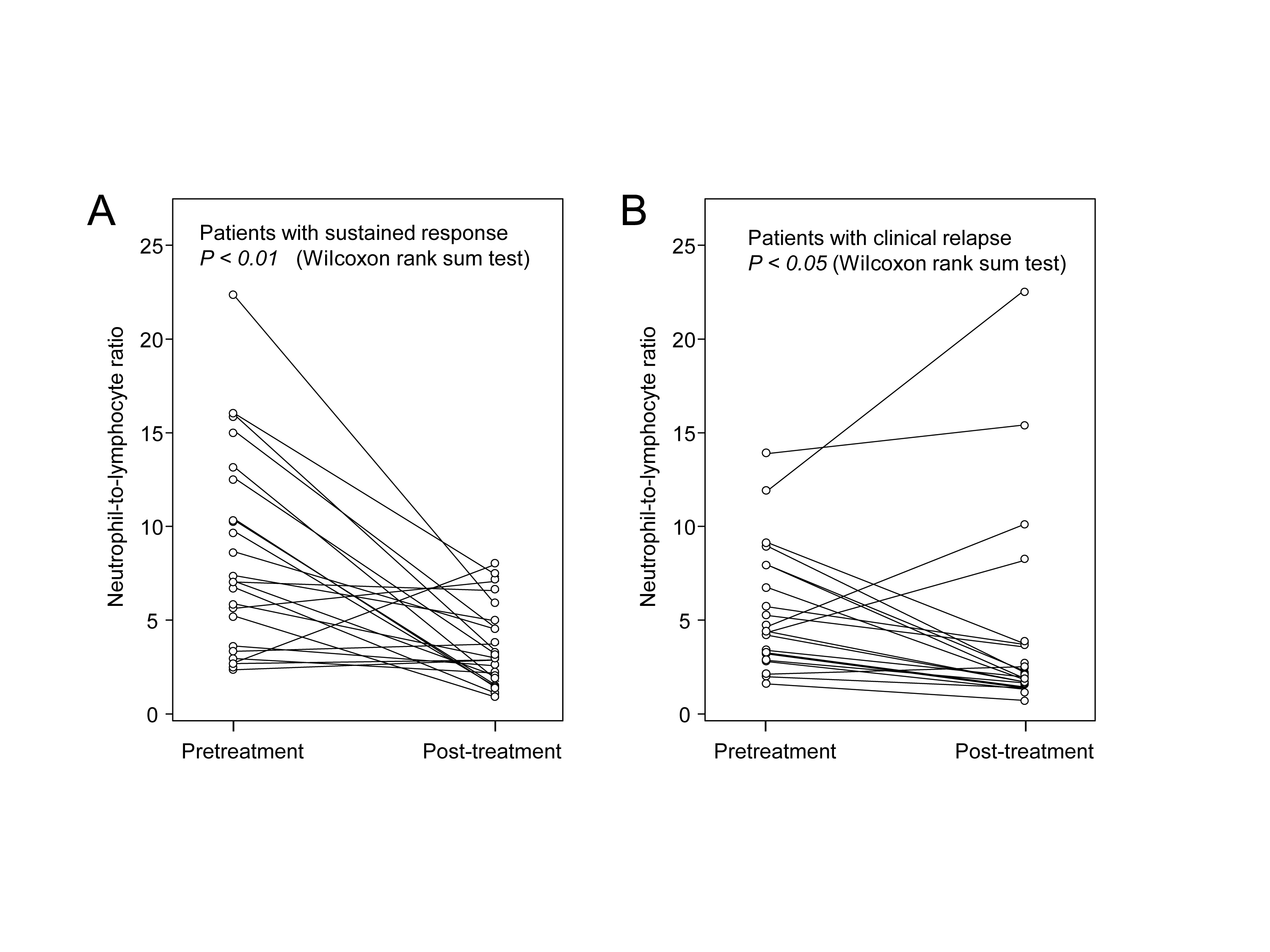

Supplement: S1 Fig — The NLR decreased after tacrolimus induction therapy in both patients with sustained response (P < 0.01) (A) and patients with clinical relapse (P < 0.05) (B) (the Wilcoxon rank sum test). (TIF) [file pone.0213505.s001.tif]

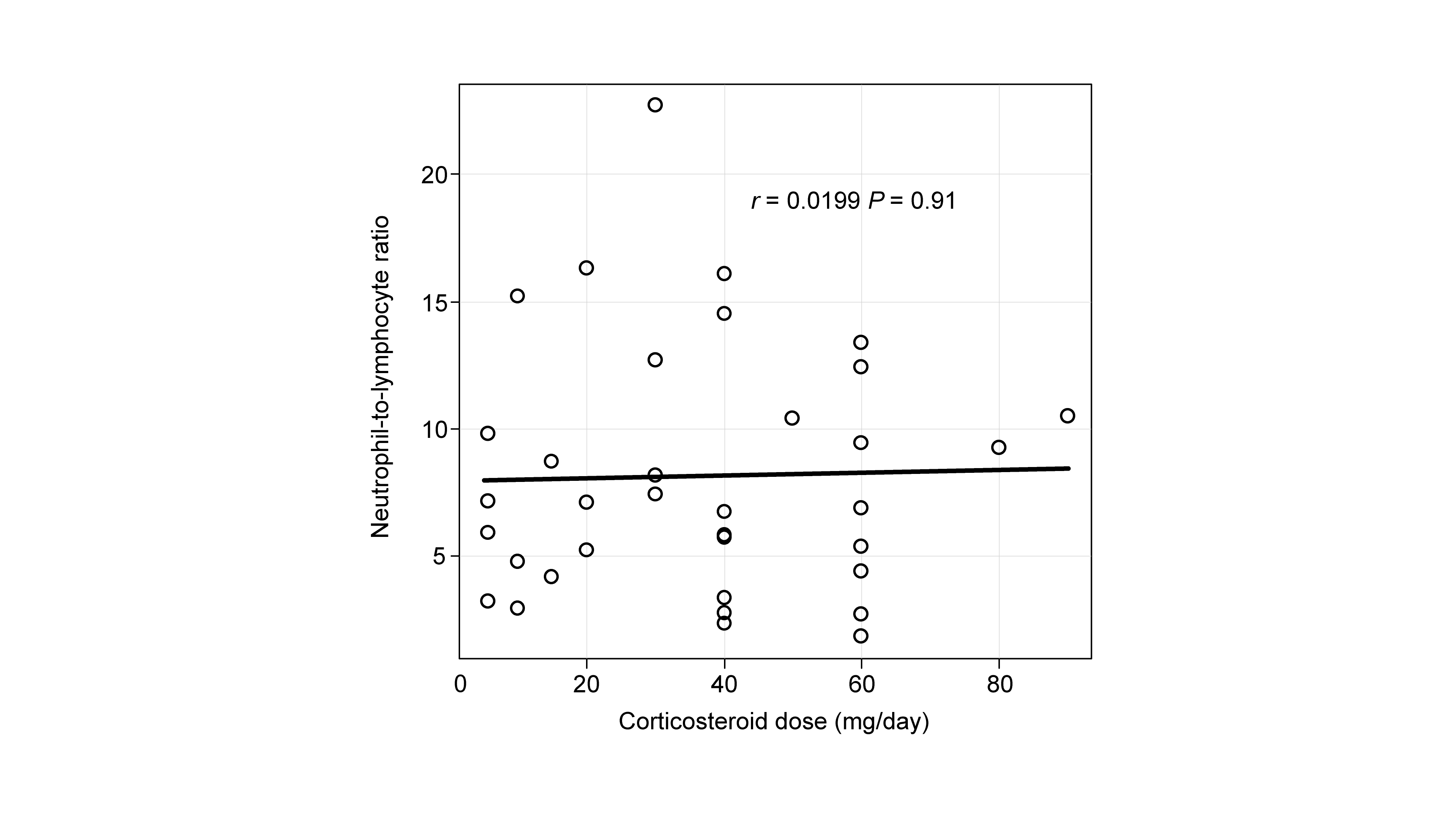

Supplement: S2 Fig — There was no correlation between corticosteroid dosage and the pretreatment NLR (r = 0.0199, P = 0.91) (the Spearman’s rank correlation). (TIF) [file pone.0213505.s002.tif]
